# Pharmacokinetic study and evaluation of the safety of taurolidine for dogs with osteosarcoma

**DOI:** 10.1186/1756-9966-32-74

**Published:** 2013-10-11

**Authors:** Kevin Marley, Stuart C Helfand, Jennifer Simpson, John E Mata, William G Tracewell, Lisa Brownlee, Shay Bracha, Bernard Séguin

**Affiliations:** 1 Department of Clinical Sciences, College of Veterinary Medicine, Oregon State University, Corvallis, OR 97331, USA; 2 Department of Biomedical Sciences, College of Veterinary Medicine, Oregon State University, Corvallis, OR 97331, USA; 3 Teva Pharmaceuticals, 145 Brandywine Parkway, West Chester, PA 19380, USA; 4 College of Osteopathic Medicine of the Pacific-Northwest, Western University of Health Sciences, 200 Mullins Drive, Lebanon, OR 97355, USA; 5 Veterinary Medical and Surgical Group, 2199 Sperry Ave, Ventura, CA 93003, USA; 6 Animal Cancer Center, 300 W Drake Rd, Fort Collins, CO 80523, USA

**Keywords:** Taurolidine, Doxorubicin, Carboplatin, Osteosarcoma, Dog

## Abstract

**Background:**

Osteosarcoma in dogs and humans share many similarities and the dog has been described as an excellent model to study this disease. The median survival in dogs has not improved in the last 25 years. Taurolidine has been shown to be cytotoxic to canine and human osteosarcoma *in vitro*. The goals of this study were to determine the pharmacokinetics and safety of taurolidine in healthy dogs and the safety of taurolidine in combination with doxorubicin or carboplatin in dogs with osteosarcoma.

**Methods:**

Two percent taurolidine was infused into six healthy dogs (150 mg/kg) over a period of two hours and blood samples were taken periodically. One dog received taurolidine with polyvinylpyrrolidone (PVP) as its carrier and later received PVP-free taurolidine as did all other dogs in this study. Serum taurolidine concentrations were determined using high-performance liquid chromatography (HPLC) online coupled to ESI-MS/MS in the multiple reaction monitoring mode. Subsequently, the same dose of taurolidine was infused to seven dogs with osteosarcoma also treated with doxorubicin or carboplatin.

**Results:**

Taurolidine infusion was safe in 6 healthy dogs and there were no significant side effects. Maximum taurolidine serum concentrations ranged between 229 to 646 μM. The dog that received taurolidine with PVP had an immediate allergic reaction but recovered fully after the infusion was stopped. Three additional dogs with osteosarcoma received doxorubicin and taurolidine without PVP. Toxicities included dilated cardiomyopathy, protein-losing nephropathy, renal insufficiency and vasculopathy at the injection site. One dog was switched to carboplatin instead of doxorubicin and an additional 4 dogs with osteosarcoma received taurolidine-carboplatin combination. One incidence of ototoxicity occurred with the taurolidine- carboplatin combination. Bone marrow and gastro-intestinal toxicity did not appear increased with taurolidine over doxorubicin or carboplatin alone.

**Conclusions:**

Taurolidine did not substantially exacerbate bone marrow or gastro-intestinal toxicity however, it is possible that taurolidine increased other toxicities of doxorubicin and carboplatin. Administering taurolidine in combination with 30 mg/m^2^ doxorubicin in dogs is not recommended but taurolidine in combination with carboplatin (300 mg/m^2^) appears safe.

## Introduction

Osteosarcoma (OS) is diagnosed in up to 1000 people per year in the United States and most of these are children and adolescents [1]. It is the most common cancer of bone in dogs and occurs at a much higher incidence [2]. In fact, conservative estimates suggest there are up to 10,000 new cases of canine OS annually in the United States [3]. The tumor biology, genetic profile and therapeutic approach to canine OS is similar to the human cancer and a number of studies have validated canine OS as a relevant model for the development of new therapeutic strategies for humans with OS [4,5]. The median survival for dogs with appendicular OS treated by amputation and adjuvant chemotherapy is 10 to 12 months and has been unchanged for the past 25 years [6]. In the present study we investigated the antimicrobial drug taurolidine as a potential adjuvant therapy for OS.

Taurolidine, a derivative of the amino acid taurine, has anti-tumor and anti-angiogenic effects against a variety of cancers [7,8]. It inhibits cellular proliferation [9] and tumor growth by inducing apoptosis [10-12], in part through p53-dependent mechanisms [13]. Taurolidine induces apoptosis by altering the Bcl-2/Bax ratio [14], and exerts anti-metastatic effects within tumor microenvironments by inhibiting angiogenesis and endothelial cell adhesion [15]. Taurolidine appears to induce autophagy and necroptosis in glioma cell lines [16] and has been used to treat glioblastoma and gastric carcinoma in people [17,18]. Taurolidine is cytotoxic to human and canine osteosarcoma cells in vitro and can be synergistic with doxorubicin or carboplatin at certain concentrations [10,19].

The goal of this study was to investigate the potential to develop taurolidine as an adjuvant treatment for OS using a canine model. The first objective was to determine the pharmacokinetic profile and safety of intra-venous (IV) taurolidine in healthy dogs. The second objective was to determine the safety of IV taurolidine in conjunction with doxorubicin or carboplatin treatments in dogs with osteosarcoma.

## Methods

### Taurolidine infusion in healthy dogs

Intravenous taurolidine was administered over a period of two hours to 6 healthy dogs at a dose of 150 mg/kg (Table 1). This dose was derived from the amount used in human trials [20,21]. The first dog received 2% taurolidine in 5% polyvinylpyrrolidone (PVP) (TauroPharm GmbH, Waldbüttelbrunn, Germany). Due to an allergic reaction to PVP, all subsequent dogs received 2% taurolidine without PVP. To make the PVP-free solution, taurolidine in powder form was dissolved in ultrapure water to a concentration of 2% W/V and sterilized in an autoclave at 121 degrees C for 30 minutes.

**Table 1:** Signalment of healthy dogs that received taurolidine for pharmacokinetic and safety study


1	Mixed breed	Female spayed	5 years old
2	Mixed breed	Male neutered	5 years old
3	Hound mix breed	Female spayed	5 years old
4	Golden retriever	Male neutered	5 years old
5	Labrador retriever	Male neutered	2 years old
6	Labrador retriever	Male neutered	3 years old

A central catheter was placed in the jugular vein for the collection of blood samples and a peripheral catheter was placed in the cephalic vein for the administration of the taurolidine. A baseline complete blood count (CBC), chemistry profile, prothrombin time (PT), partial thromboplastin time (PTT), and urinalysis (UA) were performed. A filter was placed in the IV line administering the taurolidine (Hemo-Nate, Utah Medical Products Inc., Midvale, UT, USA) and taurolidine was administered as a constant rate infusion into the catheter over a period of 120 minutes. Blood samples (5 mls each) were collected before and at 15, 30, 45, 60, 75, 90, 105, 120, 125, 150, 180 minutes, and 4, 6, 12 and 24 hours after the start of the taurolidine administration. Serum from each sample was separated by centrifugation and stored at −80°C until analysis.

For the first 3 dogs, heart rate and rhythm, respiration rate, and blood pressure were recorded before and approximately every 10 minutes for the first 3 hours following the initiation of infusion. Blood pressure was monitored using an oscillometric measurement technique (Cardell Monitor, Midmark, Tampa, Fl, USA). Heart rate and rhythm and respiration rate were recorded again 3.5, 4, 6, 12, 18, and 24 hours post infusion. Rectal temperature was monitored before and at 5, 20, 60, and 120 minutes after the start of the injection and then at 6, 12, 18, and 24 hours. Temperature, heart and respiratory rates, appetite, and general attitude were then monitored daily for 21 days and on day 28 post-infusion. A CBC, chemistry panel, and UA were performed 1, 2, 4, 7, 10, 14, 17, 21, and 28 days post-infusion. The PT and PTT tests were repeated 30 minutes after the end of each infusion. For the last 3 dogs, monitoring differed only in that the blood presure was not monitored.

### Determining serum taurolidine concentrations

Serum taurolidine concentrations were assessed using high-performance liquid chromatography (HPLC) online coupled to electrospray ionisation tandem mass spectrometry (ESI-MS/MS) in the multiple reaction monitoring mode [21]. Based on the assumption that taurolidine in aqueous solution is rapidly converted to its active metabolites taurultam and taurinamide [20], the serum from dogs treated with taurolidine was assessed for changes in taurultam and taurinamide levels during and after taurolidine infusion. Taurolidine concentrations were subsequently back-calculated as previously described [21].

Kinetic parameters for each infusion were calculated using a non-compartment model with first-order elimination (WinNonlin software, v. 4.1.a). Estimated parameters included Lambda z (1/hr), serum half-life (t1/2, hr), clearance (CL, L/hr/kg), volume of distribution at steady state (Vss, L/kg) and mean residence time (MRT, hr). Area under the curve to last quantifiable concentration (AUClast, hr*ug/mL) was estimated using the log-linear trapezoidal method. Area under the serum concentration-time profile from time zero (AUCINF, hr*ug/mL) was extrapolated from the combined area defined by AUClast plus (Clast/kel). Maximum concentration (Cmax) and time to Cmax (Tmax) were determined by observation.

### Taurolidine in dogs with osteosarcoma

Seven dogs with osteosarcoma received taurolidine in combination with doxorubicin (30 mg/m^2^) or carboplatin (300 mg/m^2^) IV (Table 2). For most treatments, the doxorubicin or carboplatin was administered first over a period of 20 to 30 minutes and was followed within 20 minutes with the taurolidine infusion, as described above. Some treatments consisted of taurolidine alone or carboplatin alone. The first two dogs developed limb edema after drug administration through the peripheral catheter. Consequently the following 4 dogs had a vascular access port (VAP, Access Technologies, Skokie, IL) placed in their jugular vein to allow IV administration of doxorubicin or carboplatin and taurolidine through a central line. In the 6th dog, the VAP did not remain patent so carboplatin and taurolidine were administered IV in different peripheral veins at each treatment and the 7th dog was treated in the same fashion. The intended dose interval was 2 weeks for the treatments including doxorubicin [22] and 3 weeks for those with carboplatin [23]. All dogs received dolasetron IV at 0.6 mg/kg the day of doxorubicin or carboplatin administration and maropitant for 4 days starting the day after receiving doxorubicin or carboplatin, which is standard of care at our institution for dogs receiving chemotherapy.

**Table 2:** Patient characteristics, treatment and side effects for dogs with osteosarcoma


Dog ID #	7	8	9	10	11	12	13
Weight (kg)	56	74	64	37	28	47	30
Breed	Great Pyrenees	Irish wolfhound	Great Pyrenees	Golden retriever	Shepherd mix	Labrador cross	Old English sheepdog
Age (yr)	6	4	6	7	11	7	5
Sex	Neutered male	Spayed female	Spayed female	Neutered male	Spayed female	Neutered male	Spayed female
Tumor location	Distal radius	Distal radius	Distal radius	Proximal tibia	Proximal humerus	Distal radius	Distal radius
Chemo drug	Doxorubicin	Doxorubicin	1 dose doxorubicin then carboplatin	Carboplatin	Carboplatin	Carboplatin	Carboplatin
Number of taurolidine treatments	4	10	4	5	3	5	2
Neutropenia	None	None	Grade 1 once^b^	Grade 1 four times	None	Grade 1 once	Grade 1 once
			Grade 2 once			Grade 2 once	
Thrombocytopenia	None	Grade 1 twice	None	None	None	Grade 2 once	None
		Grade 2 twice					
Chemistry panel	Normal	Low albumin, high ALP which returned to normal in 2 weeks	Azotemia^b^	Normal	Normal	ALT elevation^c^	Normal
Constitutional adverse event	Lethargy grade 1 to 2^a^	Grade 1 fever once	Lethargy grade 1 once	Lethargy grade 1 once	Grade 2 fever and lethargy grade 3 once, both associated with extravasation of carboplatin or taurolidine	Lethargy grade 1 twice and grade 2 twice	None
GI toxicity	Anorexia grade 2^a^	Anorexia grade 1 twice	Diarrhea grade1^b^	None	None	Anorexia grade 1 once and grade 2 twice	Anorexia grade 1 once
		Diarrhea grade 1 once					Diarrhea grade 1 once
Other adverse event or complication	Edema in limb which was used for IV infusion, vasculopathy at injection sites	Phlebitis and limb edema	Renal insufficiency^b^	None	Hearing loss	None	None
		Congestive heart failure					
		Proteinuria (urine protein/creatinine = 7.9) nephropathy					
Outcome	Euthanized due to progressive disease ^a,e^	Died of heart failure^e^	Bone metastasis documented 1071 days postoperatively^e^	Died 897 days postoperatively with pulmonary metastasis^d,e^	Euthanized 174 days postoperatively due to pulmonary metastasis^e^	Euthanized 165 days postoperatively due to metastasis to scapula^e^	Euthanized 116 days postoperatively due to metastasis to lungs, liver, and spine ^d, e^
	Necropsy performed	Necropsy performed	Still alive 1186 days postoperatively	Necropsy performed			Necropsy performed

^a^This dog had stage III osteosarcoma when the taurolidine/doxorubicin treatments were given. It was difficult to assess the number of events for the anorexia and lethargy as these adverse events were pervasive during the treatment period.

^b^Grade 1 neutropenia, diarrhea, and azotemia occurred after the doxorubicin/taurolidine combination treatment: Blood Urea Nitrogen and creatinine were as high as 60 mg/dL (normal 7–27 mg/dL) and 3.1 mg/dL (normal 0.4-1.8 mg/dL), respectively. The renal function recovered before starting the carboplatin and taurolidine treatments.

^c^ALT (alanine transaminase) with up to 371 IU/L (normal 0–113 IU/L) after the 4th taurolidine treatment and 6th carboplatin treatment. Two weeks later it was down to 129 IU/L.

^d^Dog #10 was entered in another clinical trial 270 days postoperatively where it received dasatinib and dog #13 also received bortezomib 102 days after amputation once metastatic disease was detected on chest radiographs.

^e^Confirmed with histology.

Complete blood counts were performed the day before, or the day of, and 7 to 14 days after treatments. Other monitoring included chemistry panels, urinalyses and thoracic radiographs at differing intervals. Owners were questioned about gastrointestinal toxicity and the presence of any other clinical signs at each visit. Toxic side effects were graded using the Veterinary Co-operative Oncology Group Consensus Statement [24]. All procedures were approved by the Institutional Animal Care and Use Committee of the Oregon State University. Owner’s consent was obtained for dogs with osteosarcoma.

## Results

### Pharmacokinetic and safety in 6 healthy dogs

**Figure 1:**
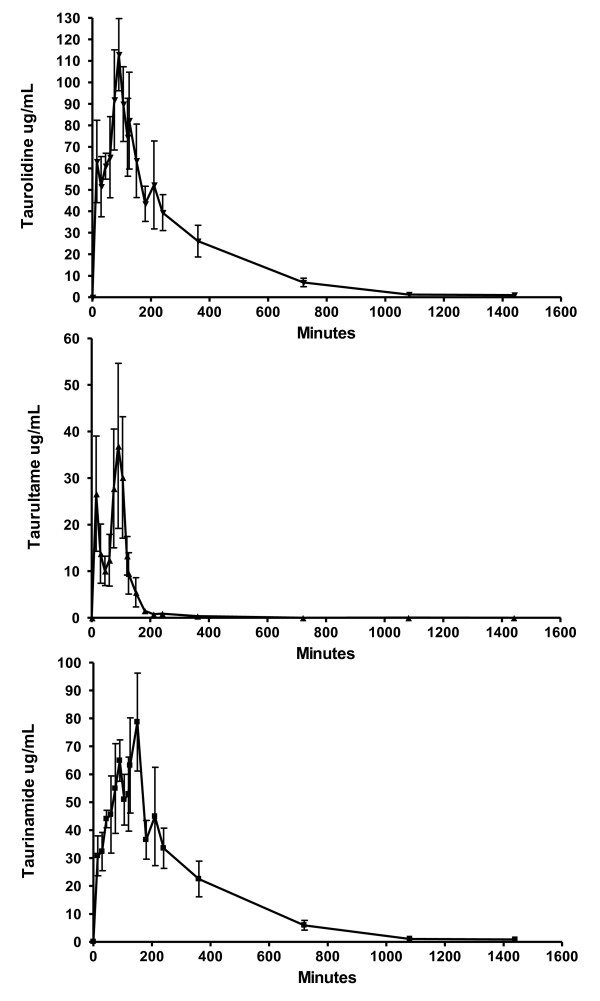
**Serum concentrations of taurolidine as calculated from the concentrations of taurultame and taurinamide (see Methods section) following a 150 mg/kg IV infusion of 2% taurolidine.**

Mean pharmacokinetic profiles of taurinamide, taurultame, and taurolidine are displayed in Figure 1. Plasma concentrations of taurolidine increased rapidly after the start of each infusion and continued to increase until the infusion was stopped, then declined rapidly and were not detectable at 18 hours post infusion. Pharmacokinetic parameters are presented in Table 3. An allergic reaction developed in healthy dog #1 within one minute of starting the infusion of taurolidine containing PVP and the infusion was stopped. The dog initially became very agitated, pawing at and shaking its head, looking disoriented, and then became sedated. Thirty-five minutes after the infusion, the dog vomited. The mucous membrane color was paler and the blood pressure dropped to a mean of 45 mm Hg 45 minutes after the infusion but the dog recovered fully after 90 minutes. All subsequent infusions used taurolidine without PVP and no such reaction was present in any dog, including healthy dog #1, when this solution was used.

**Table 3:** Pharmacokinetic parameters following a 150 mg/kg taurolidine infusion over 2 hrs

**ID**	**Cmax (ug/mL)**	**Tmax (hr)**	**Lambda_z (1/hr)**	**t1/2 (hr)**	AUClast (hr*ug/mL)	AUCINF (hr*ug/mL)	**CL (L/hr/kg)**	**Vss (L/kg)**	**MRT (hr)**
1	65.6	1.50	0.192	3.6	178	197	0.763	3.11	4.1
2	144.8	2.00	0.203	3.4	667	680	0.221	0.85	3.9
3	185.0	1.50	0.201	3.5	457	467	0.321	0.97	3.0
4	145.1	1.25	0.201	3.4	337	339	0.442	1.26	2.8
5	162.5	2.08	0.183	3.8	684	698	0.215	1.09	5.1
6	139.7	1.75	0.272	2.6	346	348	0.431	1.09	2.5
Mean	140.5	1.63*	0.209	3.4	445	455	0.399	1.39	3.6
SD	40.3	NA	0.032	0.4	200	201	0.204	0.85	0.9
CV%	28.7	NA	15.3	12.7	44.9	44.1	51.0	60.9	26.5

*median; *Cmax* Maximum concentration, *Tmax* Time to Cmax, *t1/2* serum half-life, *AUClast* area under the curve to last quantifiable concentration, *AUCINF* Area under the serum concentration-time profile from time zero, *CL* Clearance, *Vss* Volume of distribution at steady state, and *MRT* Mean residence time.

**Figure 2:**
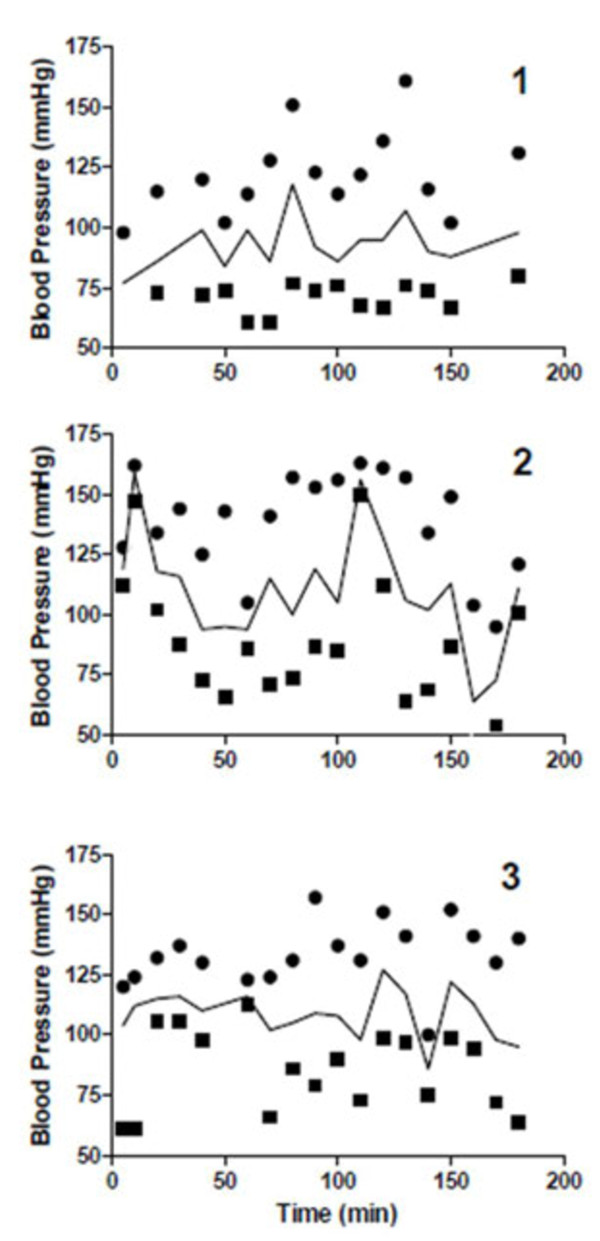
**Graphs demonstrating the blood pressures of healthy dogs 1, 2, and 3 before, during (5 to 120 minutes) and after (130 to 180 minutes) the taurolidine infusion.** ● are systolic, ■ are diastolic, and solid lines are the mean blood pressures.

There were no abnormalities in any of the body temperatures, heart rates or rhythm, and respiration rates throughout the study period in any of the dogs receiving a PVP-free solution of taurolidine. Blood pressures varied between 47–150 mm Hg, 59–159 mm Hg, and 64–163 mm Hg for the diastolic, mean, and systolic pressures, respectively (Figure 2). There were no significant abnormalities in the chemistry panels, coagulation profile tests or urinalyses in any of the dogs.

No abnormalities in CBCs were noted except dog #4 developed a neutropenia grade 1. Seven months prior to the study, a CBC was performed on healthy dog #4 and a neutropenia was present (2705/μl; normal range 3000-11400/μl). On the day of administration of taurolidine, prior to the infusion, the neutrophil count was 5510/μl. Two days later, neutropenia was present (2925/μl) and varied from then on between 1550-2925/μl. Monitoring beyond the study period (28 days) occurred in this dog. The neutrophil count at 10 and 13 weeks and 12 and 28 months after the infusion were 2340, 2558, 2536, and 2800/μl, respectively. Six weeks after the infusion (neutrophil count of 2415/μl) a bone marrow aspirate was done and showed adequate number of myeloid cells with normal progression of maturation. The myeloid to erythroid ratio was approximately 2:1 showing a mild increase in myeloid cells with respect to erythroid cells. At the time of writing, the dog remained healthy, 43 months after the infusion.

### Taurolidine in dogs with osteosarcoma

A total of 33 doses of taurolidine were administered to the clinical dogs. Four were given before initiating doxorubicin or carboplatin, 8 were combined with doxorubicin at the same treatment session, 3 were in between doxorubicin treatments, 17 were combined with carboplatin at the same treatment session, and 1 was given in between carboplatin treatments (Table 2).

Toxicity to the bone marrow and gastrointestinal system related to the administration of taurolidine with or without doxorubicin or carboplatin is presented in Tables 2 and 4. Other adverse events were (Table 2): There were 4 episodes of grade I and 2 of grade II lethargy, all occurring when combined with carboplatin. One dog had grade II lethargy throughout most of the treatment protocol but this dog had gross metastatic disease. One episode of fever grade I occurred when combined with doxorubicin. One dog developed a grade II fever, lethargy and inappetance after suspected subcutaneous injection of carboplatin or taurolidine and infection of the VAP. One dog treated with the combination with doxorubicin developed congestive heart failure which lead to its euthanasia. Renal toxicity occurred in 2 dogs when combined with doxorubicin: one dog developed azotemia and the other developed proteinuria and hypoalbunemia with renal damage detected on necropsy. Limb edema occurred in the two dogs where taurolidine and doxorubicin were injected in the same peripheral catheter. Ototoxicity manifested as hearing loss developed in one dog receiving the taurolidine and carboplatin combination.

**Table 4:** Number of events for toxicities associated with the administration of taurolidine to dogs with osteosarcoma

**Toxicity**	**Grade I**	**Circumstances**	**Grade II**	**Circumstances**
**Bone marrow**				
Neutropenia	7	1 when T combined with D	2	Both when T combined with C
		6 when T combined with C		
Thrombocytopenia	2	1 when T alone	3	2 when T combined with D
		1 when T combined with D		
				1 when T combined with C
**Gastrointestinal**				
Anorexia	4	2 when T combined with D	5	3 when T combined with D
		1 when T combined with C		2 when T combined with C
		1 when T given by itself one week after C		
Diarrhea	3	2 when T combined with D	0	
		1 when T combined with C		

T = taurolidine, D = doxorubicin, C = carboplatin.

Delay of doses or dose reductions in carboplatin or doxorubicin associated with the combination of taurolidine are presented in Table 5. In this study, five doses of carboplatin were administered without taurolidine and three resulted in grade I neutropenia, one of which resulted in a dose delay.

**Table 5:** Delay of doses or dose reductions in carboplatin or doxorubicin associated with the combination of taurolidine

**Dose delay**	**Case #**	**Caused by taurolidine combined with**	**Number of times for delay or reduction**	**Reason/circumstances**
	10	Carboplatin	2	Neutropenia
			1	Had been neutropenic previous 2 times so delayed dose without checking neutrophils at 21 days post treatment
	9	Doxorubicin	1	Renal insufficiency
	7	Doxorubicin	1	Lethargy and inappetance
	8	Doxorubicin	1	Limb edema
	12	Carboplatin	1	Neutropenia: taurolidine had been given 9 days after the carboplatin treatment
	9	Carboplatin	1	Owner’s restrictions
	11	Carboplatin	1	
	12	Carboplatin	1	
Dose reduction				
	7	Doxorubicin	2	Lethargy and inappetance throughout chemotherapy period
	9	Doxorubicin	4	Dose reduction was done for the all carboplatin treatments due to the renal insufficiency that the dog had developed after the combination with doxorubicin
	10	Carboplatin	1	Neutropenia

## Discussion

### Pharmacokinetic and safety in normal dogs

The pharmacokinetic parameters achieved in dogs were comparable to those in humans (Table 6). This justifies the dose of 150 mg/kg used in dogs in this study. Because higher levels of taurolidine were achieved in dogs, the dose in dogs could be decreased if the goal is to achieve same levels as in humans. There is also a 300 mg/kg dose that is reported in humans [8,17] which in all likelihood leads to higher concentrations of taurolidine than is reported in the pharmacokinetic studies that used a dose of about 75 mg/kg [20,21]. Although the human values are only estimations based on a 66 kg body mass, several differences between dog and human are noteworthy. Dogs appear to have a smaller Vss than humans with longer t1/2, CL and AUC which provides some explanation for the higher levels of taurolidine achieved in dogs.

**Table 6:** Comparison of pharmacokinetic parameters in dogs and humans after taurolidine infusion

**Parameter**	**Dog**	**Human**
C_max_ (μg/mL)	140.5 ± 40.3	95.8 ± 21.8
t_1/2_ (hr)	3.4 ± 0.4	1.7 ± 0.1
AUC (μg h/mL)	455 ± 200	249.2 ± 58.4
CL (L/hr/kg)	0.40 ± 0.20	1.28*
Vss (L/kg)	1.39 ± 0.85	3.18*

*Estimated in humans based on a 66 kg body weight from the data published by Stendel et al. 2007. Numbers in humans are after 3 infusions of taurolidine, 2 hours long each with a one hour interval between [21] whereas the numbers in dogs are after a single 2 hour long infusion.

Healthy dog#1 achieved a considerably lower Cmax and Area Under the Curve than all other dogs. It is unknown why this was the case. Pharmacokinetic studies done on a larger number of dogs are necessary to try to answer this question.

Healthy dog #1 also manifested an allergic-like reaction within a minute of starting the infusion of taurolidine containing PVP. The reaction was attributed to the PVP. The dog received a total dose of 6.5 mg/kg of PVP. PVP injected in dogs causes immediate release of histamine systemically and at high dose (100 mg/kg) prolonged hypotension [25-27]. In the dose range of PVP that the dog in our study received, the response in dogs is variable but can include labored respiration, unsteady gate, shaking and scratching of the head [27]. The clinical signs manifested in the dog of this study were partly similar in nature (agitation, pawing at and shaking its head, looking disoriented). This same dog, and all other dogs in this study, did not have this type of reaction or any other signs of an allergic reaction when given the PVP-free solution.

Because of the allergic reaction to PVP in dogs, we had to administer a PVP-free solution of taurolidine. PVP increases the stability of taurolidine in aqueous solution [28]. In aqueous solution taurolidine breaks down into and becomes in equilibrium with taurultame and taurinamide which eventually leads to the release of formaldehyde. PVP helps to push the equilibrium towards taurolidine such that in the presence of PVP, there is less formaldehyde in solution [28]. The therapeutic implications of a PVP-free solution of taurolidine are unknown. It is not determined which molecule exerts the therapeutic benefit of taurolidine between taurolidine itself, taurultame, taurinamide or formaldehyde. In our *in vitro* experiments, PVP-free taurolidine was effective at killing OSA cells below the serum concentrations achieved in the healthy dogs [19].

Healthy dog #4 had neutropenia following the infusion of taurolidine. Whether the taurolidine played a role in the presence of neutropenia is difficult to ascertain. Seven months before the infusion, the dog was mildly neutropenic when comparing to the normal range in our laboratory. Also the neutropenia observed after the infusion of taurolidine was very mild (grade 1) so it may be that this dog was normally waxing and waning around the low end of the normal range, mostly running at a lower neutrophil count than the normal range. A bone marrow aspirate determined that the lower neutrophil count was not due to bone marrow suppression. In a study in rats, the administration of taurolidine did not affect leucopoiesis [29]. The dog was monitored daily for over 3.5 years after the administration of taurolidine and remained healthy. It is unlikely that taurolidine had an effect on the neutrophil count of this dog.

There is no consensus on what blood pressure value constitutes hypertension in dogs [30,31]. It has been suggested that hypertension occurs when systolic blood pressure is ≥ 150 to 180 mm Hg. Systolic blood pressure in normal dogs when measured by oscillometric method has been reported to vary between 131 to 150 ± 20 mm Hg [31]. Therefore 150 mm Hg appears a low cut off value to diagnose hypertension. When choosing a cut off value of 180 mm Hg, none of the dogs had episodes of hypertension. If based on the cutoff value of 150 mm Hg, there were several episodes of transient hypertension. This could very well be physiologic and related to stress [30,31]. Even in the event that these episodes of hypertension were related to the administration of taurolidine, the hypertension was transient and the dogs did not suffer any ill consequences in the short or long term. Hypotension has been defined as a mean arterial pressure <60 mmHg [32-34]. Dog #2 had a transient episode of hypotension during the infusion (Figure 2).

### Dogs with osteosarcoma

The most common side effect of taurolidine infusion in humans is a local reaction manifested by burning at the infusion site, numbness or soreness of the infusion arm, and erythematous striking at the IV site. Twenty-eight percent of subjects had facial flushing during the infusion and 5% each for headache, epistaxis, and nausea [20]. None of the healthy dogs appeared to be bothered by the taurolidine infusion in their peripheral vein. The two dogs that received doxorubicin and taurolidine in the same peripheral vein during the same treatment sessions developed limb edema. One dog had a vasculopathy at necropsy but not the other. It was hypothesized after these 2 dogs that the administration of doxorubicin and taurolidine in the same vein caused too much irritation and therefore it was decided to administer the treatments through a central line. For this, a VAP was placed in the jugular vein in the following dogs except dog #13. However, because the VAP became non-functional in dog #12, the carboplatin and taurolidine were administered in different peripheral veins and the dog did well. Based on this, the carboplatin and taurolidine administrations were given in different peripheral veins at each session in dog #13 and this dog also did not show any adverse effects at the injection sites. We suspect that injecting the combination of doxorubicin and tauroldine is not feasible in the same peripheral vein, at least not on the same day. It is unknown if carboplatin and taurolidine can be injected in the same vein during the same treatment session.

Cytopenias experienced by dogs with osteosarcoma in this study were comparable to those reported with carboplatin alone. In a study of 65 dogs with osteosarcoma treated with carboplatin alone, 20% of the carboplatin cycles lead to neutropenia grade I, 3% to grade II, and 1% to grade III; Eleven percent of the carboplatin cycles lead to thrombocytopenia grade I, 12% to grade II, and 4% grade III [23]. In this study, 7 episodes of neutropenia and one episode of thrombocytopenia were recorded after 18 combined doses of taurolidine and carboplatin. None of the cytopenias in this study were life-threatening as 5 neutropenic episodes were grade I and 2 were grade II and the one thrombocytopenic episode was grade II. Moreover, 3 out 5 doses of carboplatin administered alone resulted in grade 1 neutropenia. It is possible that the cytopenias in this study were underestimated because of the timing of the blood draws after a treatment. The nadir neutrophil and platelet counts occur on day 14 in dogs receiving carboplatin alone [35]. In our study, when the blood draws were done on days 7–13, it is possible that the neutrophil and platelet counts were normal but later dropped below normal levels. In another study 76% of the neutropenic episodes were recorded between days 18 and 22 after a carboplatin treatment [23]. In our study 4 of the 7 neutropenic episodes were recorded 21 to 29 days post taurolidine and carboplatin treatment.

In one study 24% of dogs with osteosarcoma experienced at least one episode of gastrointestinal toxicity with carboplatin alone [36]. In another, 4% of the cycles of carboplatin resulted in grade I vomiting, 2% in grade II, and <1% in grade III. Five percent of the cycles of carboplatin resulted in grade I diarrhea, 2% in grade II, and <1% in grade III [23]. The gastrointestinal toxicity experienced in the clinical dogs in this study compares favorably with these numbers.

Cardiac toxicity is a well-known and documented side effect of doxorubicin. The toxicity is cumulative [37]. In one of the largest studies in dogs reporting on the use of doxorubicin for the treatment of osteosarcoma, 8% developed heart disease [22]. In another recent study of 94 dogs with various neoplasms treated with doxorubicin, the incidence of cardiotoxicity was 8% [38]. The dog in our study that developed congestive heart failure was an Irish Wolfhound and the cardiac failure was clinically diagnosed after a cumulative dose of 90 mg/m^2^ of doxorubicin. Irish Wolfhound is a breed predisposed to developing dilated cardiomyopathy with a prevalence to be reported as high as 24% [39]. In the case in this study, a complete evaluation of the heart to include ECG and echocardiogram before starting the combination treatments was performed and found to be completely normal. Dilated cardiomyopathy was diagnosed at necropsy. It is not possible to tell if the dilated cardiomyopathy was a result of doxorubicin toxicity or idiopathic in an Irish wolfhound. On one hand the time frame to develop the dilated cardiomyopathy with congestive heart failure seems short (6 weeks from initiating doxorubicin therapy) but on the other hand the cumulative dose of doxorubicin (90 mg/m2) was relatively low for cardiotoxicity. It may very well be a combination of using doxorubicin in a high risk breed. Whether the taurolidine played any role in promoting the cardiotoxicity of doxorubicin is unknown but must be considered.

Renal toxicity caused by doxorubicin is known to occur in rabbits, pigs, and cats but appears to be very rare in dogs [37,40,41]. Rabbits treated with doxorubicin developed azotemia, proteinuria, and hypoproteinemia [42]. In this study, two dogs that were administered doxorubicin with taurolidine developed nephropathies (dogs #8 and 9). One dog developed proteinuria and hypoalbuminemia. The other dog developed renal insufficiency. In the dog with renal insufficiency, the damage was reversible as the ability to concentrate recovered. The first dog had a necropsy which revealed severe thickening of the Bowman’s capsule, mesangial matrix, and the presence of occasional proteinaceous casts in the tubules. Reported changes with doxorubicin alone in cats, rabbits, and pigs include tubular casts, tubular dilation, stromal sclerosis, glomerular sclerosis and vacuoles, thickening of Bowman’s capsule, and thickened mesangial matrix [40,41,43]. The mechanism of nephrotoxicity with doxorubicin remains unknown [42]. Given that two of the 3 dogs that received doxorubicin with the taurolidine developed nephropathies and this is rare in dogs with doxorubicin alone, the possibility exists that taurolidine may potentiate the nephrotoxic effects of doxorubicin in this species.

To the authors’ knowledge, ototoxicity has not been reported with carboplatin in dogs. In children, ototoxicity has been reported in 4 to 10% of patients after receiving carboplatin [44,45]. It is possible that ototoxicity in the form of hearing loss occurs more frequently than we realize in dogs given the difficulty to assess this function in this species [46]. But given the rarity of ototoxicity of carboplatin in dogs, it is possible that taurolidine had a role in potentiating in dog #11 this known toxicity in humans.

Although not a goal of this study, 1 of the 5 dogs that received carboplatin in combination with taurolidine was alive at the time of writing, 1186 days postoperatively, with bone metastases diagnosed 1071 days postoperatively (dog #9) (Table 2). One dog (#10) died 897 days post amputation with pulmonary metastasis. This dog also received another investigational drug starting 270 days post amputation. The other 3 dogs were euthanized 174 (dog #11), 163 (dog #12), and 116 (dog #13) days post removal of the tumor. All 3 dogs euthanized had one or more negative prognostic factors. In dog #11, the tumor was located in the proximal humerus, the anatomic location with poor prognosis in the appendicular skeleton [36,47]. Dog #12 had a tumor that involved 82% of the length of the radius and a high mitotic index (over 21/3 high power fields [HPF]). Dog #13 had a large tumor volume with a high mitotic index (15/3 HPF). Large volume [48,49], percent of radius affected (>29% affected) [50], and mitotic index (>5/3 HPF) [22,23,51] have been reported as negative prognostic factors for disease-free interval or survival. In 2 studies on prognosis when using carboplatin alone in dogs with OS, the median disease-free interval was 256 and 137 days and median survival was and 307 and 277 days [23,36].

A recent study found that taurolidine enhanced metastasis of osteosarcoma to the liver and lungs in mice and was toxic to the liver [52]. In the present study, there is no evidence of taurolidine being toxic to the liver. One dog with OS (#12) showed an elevation in alanine transaminase (ALT) after the 4th taurolidine treatment and 6th carboplatin treatment (371 IU/L [normal range 0–113 IU/L). Two weeks later it was down to 129 IU/L. Two dogs with OS (#9 and 11) had an elevated alkaline phosphatase (ALP) or ALT before receiving taurolidine which returned to normal after receiving the combination treatments. Four healthy dogs (#1, 3, 4, and 6) had an elevated liver parameter (either ALP, ALT, or gamma-glutamyl transpeptidase [GGT], or combination of) before receiving taurolidine and the parameters either returned to normal or decreased after the taurolidine infusion. Furthermore, in the 3 dogs where a necropsy was performed, there was no evidence of liver damage. The doses of taurolidine that caused liver damage in mice were 750 mg/kg and 500 mg/kg. Based on allometric scaling of cancer drugs proposed by Freireich, et al. (1966) the equivalent dose of a drug in a dog is 1/6 the dose in a mouse [53]. This translates to the equivalent of 125 mg/kg and 83 mg/kg in a dog. The dose used in our study (150 mg/kg) was higher than the one used in the study in mice if proportions are maintained across species.

It is not possible to tell if taurolidine enhanced metastasis of osteosarcoma to the lungs and liver in the dogs of this study. Metastases to the lungs are the most common with osteosarcoma and are present in over 90% of dogs over the course of the disease [2]. In this study, two dogs died with pulmonary metastasis following treatment with taurolidine. Metastasis to the liver has been reported in dogs [54]. In this study one dog was found to have metastasis to the liver on necropsy. An important distinction between this study and the one in mice where taurolidine enhanced the pulmonary and liver metastasis is that dogs in our study were also administered either doxorubicin or carboplatin.

## Conclusions

The pharmacokinetic profile of taurolidine in dogs is similar to that reported in humans. Taurolidine is well tolerated in dogs and can be given with carboplatin. The toxicity to the bone marrow and gastro-intestinal tract was not substantially increased by taurolidine. It is possible that taurolidine exacerbates other toxicities of doxorubicin and carboplatin and this will have to be closely monitored in a larger clinical trial. At this point in time we do not recommend giving doxorubicin (30 mg/m^2^) in combination with taurolidine in dogs because of the significant side effects documented in this study with this particular combination. A larger clinical trial will also be necessary to determine if taurolidine prolongs survival in dogs with osteosarcoma.

## Abbreviations

OS: Osteosarcoma; IV: Intra-venous; PVP: Polyvinylpyrrolidone; CBC: Complete blood count; PT: Prothrombin time; PTT: Partial thromboplastin time; UA: Urinalysis; VAP: Vascular access port; HPLC: High-performance liquid chromatography; ESI-MS/MS: Electrospray ionisation tandem mass spectrometry; T1/2: Serum half life; CL: Clearance; Vss: Volume of distribution at steady state; MRT: Mean residence time; AUClast: Area under the curve to last quantifiable concentration; AUCINF: Area under the serum concentration-time profile from time zero; Clast: Last observed quantifiable concentration; kel: Elimination rate constant; Cmax: Maximum concentration; Tmax: Time to maximum concentration; HPF: High power fields; ALT: Alanine transaminase; ALP: Alkaline phosphatase; GGT: Gamma-glutamyl transpeptidase.

## Competing interests

The authors declare that they have no competing interests.

## Authors’ contributions

KM analyzed and interpreted data, and drafted the manuscript. SCH helped design the experiments, and data collection, analysis and interpretation. JS carried experiments, analyzed and interpreted data. JEM helped with experiment design and data analysis and interpretation. WGT analyzed and interpreted data. LB helped design the experiments, analyzed and interpreted data. SB helped with data collection and interpretation. BS was responsible for conception of study, design of experiments, interpretation of data and revision of manuscript for intellectual content. All authors read and approved the final manuscript.
